# Light Scattering Sensor for Direct Identification of Colonies of *Escherichia coli* Serogroups O26, O45, O103, O111, O121, O145 and O157

**DOI:** 10.1371/journal.pone.0105272

**Published:** 2014-08-19

**Authors:** Yanjie Tang, Huisung Kim, Atul K. Singh, Amornrat Aroonnual, Euiwon Bae, Bartek Rajwa, Pina M. Fratamico, Arun K. Bhunia

**Affiliations:** 1 Molecular Food Microbiology Laboratory, Department of Food Science, Purdue University, West Lafayette, Indiana, United States of America; 2 School of Mechanical Engineering, Purdue University, West Lafayette, Indiana, United States of America; 3 Bindley Bioscience Center, Purdue University, West Lafayette, Indiana, United States of America; 4 USDA-ARS, Eastern Regional Research Center, Wyndmoor, Pennsylvania, United States of America; 5 Department of Comparative Pathobiology, Purdue University, West Lafayette, Indiana, United States of America; State Key Laboratory of Pathogen and Biosecurity, Beijing Institute of Microbiology and Epidemiology, China

## Abstract

**Background:**

Shiga-toxin producing *Escherichia coli* (STEC) have emerged as important foodborne pathogens, among which seven serogroups (O26, O45, O103, O111, O121, O145, O157) are most frequently implicated in human infection. The aim was to determine if a light scattering sensor can be used to rapidly identify the colonies of STEC serogroups on selective agar plates.

**Methodology/Principal Findings:**

Initially, a total of 37 STEC strains representing seven serovars were grown on four different selective agar media, including sorbitol MacConkey (SMAC), Rainbow Agar O157, BBL CHROMagarO157, and R&F *E. coli* O157:H7, as well as nonselective Brain Heart Infusion agar. The colonies were scanned by an automated light scattering sensor, known as BARDOT (BActerial Rapid Detection using Optical scattering Technology), to acquire scatter patterns of STEC serogroups, and the scatter patterns were analyzed using an image classifier. Among all of the selective media tested, both SMAC and Rainbow provided the best differentiation results allowing multi-class classification of all serovars with an average accuracy of more than 90% after 10–12 h of growth, even though the colony appearance was indistinguishable at that early stage of growth. SMAC was chosen for exhaustive scatter image library development, and 36 additional strains of O157:H7 and 11 non-O157 serovars were examined, with each serogroup producing unique differential scatter patterns. Colony scatter images were also tested with samples derived from pure and mixed cultures, as well as experimentally inoculated food samples. BARDOT accurately detected O157 and O26 serovars from a mixed culture and also from inoculated lettuce and ground beef (10-h broth enrichment +12-h on-plate incubation) in the presence of natural background microbiota in less than 24 h.

**Conclusions:**

BARDOT could potentially be used as a screening tool during isolation of the most important STEC serovars on selective agar plates from food samples in less than 24 h.

## Introduction

Shiga-toxin producing *Escherichia coli* (STEC) strains are recognized as serious foodborne pathogens and comprise of a diverse group of organisms, related to their O-group designations and virulence gene profiles. Although STEC O157 is the most widely recognized, other serogroups have been increasingly implicated in cases of foodborne human diseases [1]–[4]. The proportion of illnesses linked to non-O157 STEC (%  =  number of non-O157/total STEC infections) is estimated to be 10–80%, but the percentages differ greatly based on geographical areas, which range from 30–80% in European countries [5]–[8] and 50–63% in North America [9], [10]. The most common non-O157 STEC serogroups identified as causes of human infections include O26, O45, O103, O111, O121 and O145 [11], [12]. Foods from which non-O157 STEC have been isolated include sausage, ice cream, milk, lettuce, and cucumber [13]–[17]. In 2011, the US Department of Agriculture Food Safety and Inspection Service (USDA-FSIS) declared the presence of these serogroups in beef trimmings to be adulterants, and testing for these serogroups became effective in June 2012 [18], [19]. These ongoing events serve as a constant reminder for the need of reliable, user-friendly, and low cost screening tools for major STEC strains to prevent outbreaks.

Use of traditional cultural methods utilizing selective and chromogenic differential media is still considered a “gold standard” for isolation and detection of STEC, regardless of whether or not other immunological or/and molecular typing methods are employed in the testing protocol. Sorbitol MacConkey (SMAC) agar supplemented with or without cefixime-tellurite (CT) was formulated to exploit the fermentative features of *E. coli* serotypes that result in chromogenic differentiation of most O157 from non-O157 serovars [20], [21]. While most O157 colonies are sorbitol negative and appear colorless, non-O157 serotypes appear as bright pink to mauve, making it difficult to distinguish non-O157 STEC from non-pathogenic *E. coli* based on color alone. Furthermore, sorbitol fermenting O157 variants also exist [22], and thus this property cannot be used as a firm diagnostic trait. A similar differential medium, R&F *E. coli* O157:H7 (R&F Laboratories, West Chicago, IL, USA), was designed to increase the specific isolation of both sorbitol-positive and negative O157 by introducing more fermentable carbohydrate sources [23]. However, differentiation of non-O157 STEC still remains a challenge. Other chromogenic media have been introduced, allowing color-based differentiation of non-O157 STEC serogroups, such as CHROMagar O157, Rainbow Agar and many other media developed by various academic laboratories [24]–[27]. Although these chromogenic media provide optimum recovery, improved selectivity, sensitivity, and overall accuracy for presumptive identification of most serogroups, some remain undistinguishable, for example, O157 and O111 both appear gray on Rainbow agar. Moreover, bacteria may need to be grown for 18–24 h or longer before the colony pigments are fully developed for differentiation.

Various analytical methods have been combined with selective and differential plating media for accurate detection and identification of the major serovars of STEC. Multiplex polymerase chain reaction (mPCR) methods have been widely used that target the *wzx* and other genes that are involved in encoding for O-antigen gene cluster-related proteins [28]. Recently, a DNA microarray [29] and Luminex microbead–based suspension array [30] were used to detect O-antigen gene clusters of *E. coli* serogroups (O26, O45, O91, O103, O104, O111, O113, O121, O128, O145, and O157) or an antibody microarray [31] was used to detect the organisms. These methods are laborious and costly when large amounts of samples are to be tested thus may have limitations for rapid high throughput screening applications. More recently, research suggested that microscopic variations in surface antigens can result in macroscopic differences in comprehensive colony morphology [32]–[34]. Therefore, it would be highly valued if colony morphology could be captured as a differentiator for various *E. coli* O-antigenic groups. Recently, a hyperspectral imaging method has been described for differentiation of STEC on agar plates that requires pure cultures of each test organism in separate plates. This method captures the spectral reflectance using visible-near infrared of all the collective colonies in the entire plate containing pure cultures and is used for differentiation of non-O157 STEC serovars [35]. This method is not suitable for detecting individual colonies present in a mixture on the same plate as is performed by classical microbiological testing.

**Figure 1:**
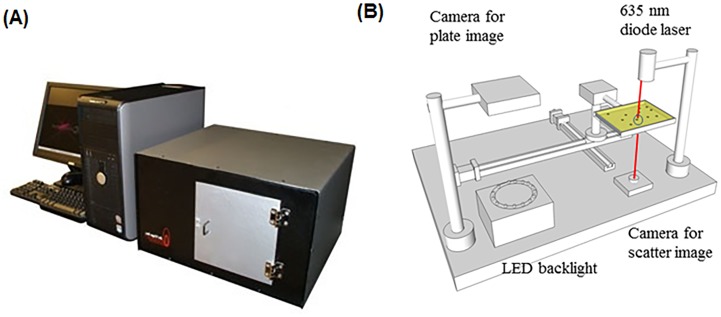
(A) The complete setup of the light scattering device, BARDOT (BActerial Rapid Detection using Optical scattering Technology). (B) Major components of the BARDOT system.

Our group has designed and built a novel laser-based light scattering device (**Fig 1**) that detects/identifies a single target colony in the presence of a mixture of colonies formed by other bacterial species and can be easily integrated with traditional culture-based methods [36]. By shining a laser (635 nm) on the center of a colony, this instrument generates scatter patterns in a 2-dimensional plane, which is mathematically expressed as Fourier transform of the input aperture field that is dependent on the macromolecular composition/structure such as shape, chromogen, and colony composition (O-antigen, peptidoglycan or metabolic by-products) to produce species or serovar-specific scatter signatures [37]–[39]. This method is label-free and does not require any specialized reagents or antibodies, but requires a scatter image library for detection or identification of an unknown organism. Thus, this system has the potential for high throughput screening of multiple microorganisms if grown on the same plate. Furthermore, since laser scatter patterns are used as an identification fingerprint, minute structural changes in the single cell is amplified in the colony morphotype, allowing for automated classification of serovars differentiated by metabolic activities and surface carbohydrate profiles.

Our previous research demonstrated that the light-scattering system is able to differentiate colonies of *E. coli* O157:H7 from bacteria belonging to other genera such as *Listeria*, *Salmonella*, *Vibrio*, *Staphylococcus*, etc. [38]. In addition, BARDOT was able to detect and identify different species within a genus or different serovars within a species. For example, BARDOT was used for detection of different species of *Listeria* [37], [38], *Vibrio* [40], and the top 20 *Salmonella* serovars [41]. The objective of this study was to evaluate the application of the BARDOT for real-time detection and differentiation of colonies of the top seven STEC serogroups (O26, O45, O103, O111, O121, O145 and O157) on selective and differential chromogenic media based on optical scatter patterns.

## Results

### Selection of appropriate media for differentiation of STEC serovars using the light scattering sensor, BARDOT

**Figure 2:**
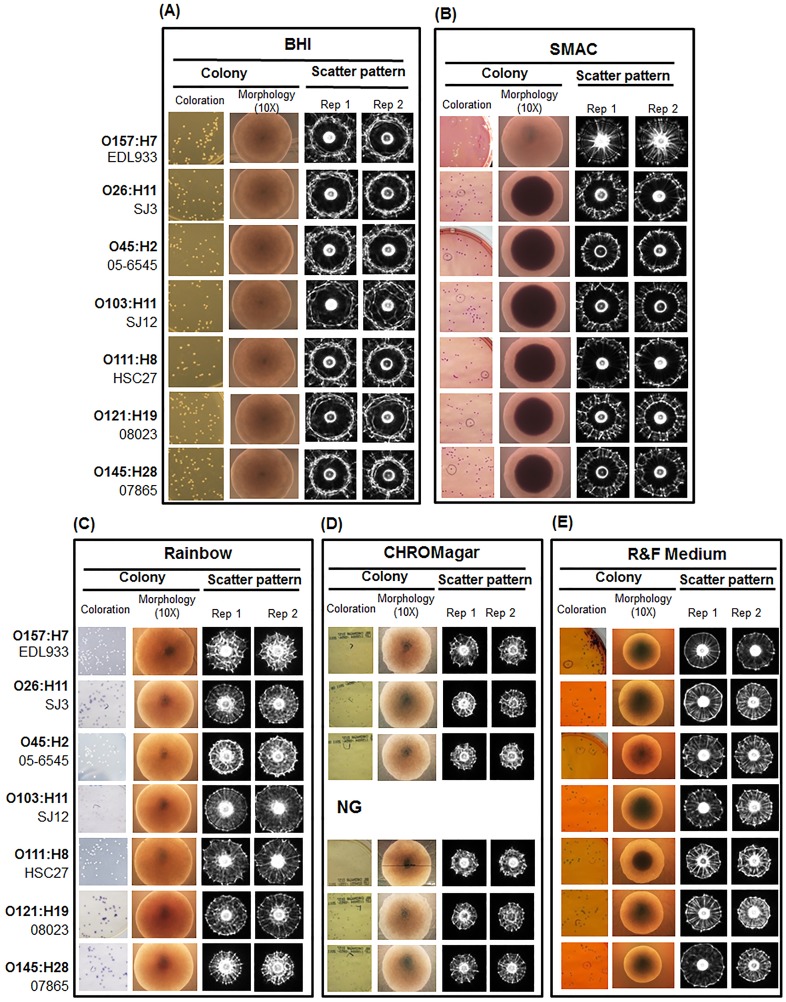
Representative images of colony coloration, microscopic colony morphology and scattering patterns of *E. coli* strains grown on each of the following medium: (A) BHI agar, (B) Sorbitol MacConkey agar (SMAC), (C) Rainbow, (D) CHROMagar O157, (E) R&F medium. All colony scatter images were captured at 10–12 h of incubation when colonies reached optimum size. ^*^NG: no growth of O103 strains on CHROMagar.

BARDOT (**Fig 1**) uses a laser to generate scatter patterns of bacterial colonies of about 1 mm diameter on agar plates for detection and identification of bacteria at the genus, species, and even at the serovar levels [38]. In this study, our initial goal was to identify a solid agar medium that would yield suitable colony scatter patterns to differentiate seven serovars of STEC (O26, O45, O103, O111, O121, O145 and O157) with high accuracy (**Table 1**). We examined commercial chromogenic differential agar media including CHROMagar O157, R&F *E. coli* O157:H7, Rainbow Agar O157 and sorbitol MacConkey (SMAC), as well as BHI agar that is nonselective. The colony morphology and scatter patterns of a representative strain from each serovar were compared on different media and each serovar produced scatter patterns that were visually distinct (**Fig 2**). With further image analysis using the accompanied software, the accuracy of discrimination among the seven serovars was represented by positive precision values (PPV) ranging from 0.91–1.0 for SMAC and 0.89–0.98 for BHI (**Table 2**). The colony scatter patterns on Rainbow and R&F media were similar; however, the signal intensity was relatively lower on R&F, owing to the accumulation of a black precipitate in the center of the colony. The PPV for Rainbow was 0.89–0.97 and for R&F 0.81–0.97 (**Table 2**). Colonies on CHROMagar formed relatively smaller diameter scatter patterns with less distinctive concentric ring structures and the PPV ranged from 0.86 to 0.94. The major drawback was that CHROMagar did not support the growth of serovar O103. This is not surprising since this medium was primarily formulated for isolation of serovar O157. Likewise, R&F *E. coli* O157:H7 was also formulated for O157:H7, thus some non-O157 serovars or strains may grow poorly or not grow at all. To further evaluate its utility for BARDOT-based detection, we excluded the antibiotic supplement to allow growth of all test serovars. Even though growth on R&F agar resulted in reasonably good discrimination (PPV  = 0.81–0.97) among the seven serogroups, this agar was not examined further. Lack of the antibiotic supplement in R&F medium may obscure STEC detection due to overgrowth of background natural microbiota when tested with real-world samples. Likewise, BHI was not selected for obvious reasons since this non-selective medium will not be able to prevent overgrowth of background microbiota during real-world food sample testing. After taking all factors into consideration, it was determined that the performance of the selective media for BARDOT-based detection was SMAC>Rainbow>CHROMagar>R&F. We chose SMAC for future studies, though other media could be used in parallel to increase confidence in STEC identification. SMAC has been widely used for selective isolation of STEC from food and has been recommended by the FDA Bacteriological Analytical Manual [42], and thus, it could be used with the BARDOT system without altering established methodology in a testing laboratory.

**Table 1:** Shiga toxin-producing *Escherichia coli* strains used in this study.

Serogroup	Serotype	Strain	Source^a^	Virulence Genes		
				*stx_1_*	*stx_2_*	*eaeA*
O157	O157:H7	EDL933	Our collection	+	+	+
	O157:H7	SEA 13A53	Our collection	+	+	+
	O157:H7	SEA 13A72	Our collection	−	+	+
	O157:H7	O1	Our collection	+	+	+
	O157:H7	G5244	Our collection	+	+	+
O26	O26:H11	90.0105	Our collection	+	−	+
	O26:H11	SJ3	P. Fratamico	−	+	+
	O26:H11	00971	P. Fratamico	+	−	+
	O26:H11	05-6544	P. Fratamico	+	−	+
	O26:H11	93-3118	P. Fratamico	+	−	−
	O26:H11	94-0962	P. Fratamico	NT^b^	NT	NT
O45	O45:H2	SJ9	P. Fratamico	+	+	+
	O45:H2	05-6545	P. Fratamico	+	−	+
	O45:H2	SJ7	P. Fratamico	+	−	+
	O45:H2	SJ8	P. Fratamico	+	−	+
	O45:H2	96-3285	P. Fratamico	+	−	+
O103	O103:H2	87.1368	Our collection	+	−	−
	O103:H6	04162	P. Fratamico	+	−	+
	O103:H11	SJ12	P. Fratamico	+	−	+
	O103:H11	04-3973	P. Fratamico	+	−	+
	O103:H2	90-3128	P. Fratamico	+	−	+
O111	O111:H8	HSC27	Our collection	+	+	+
	O111:NM	SJ13	P. Fratamico	+	+	+
	O111:H8	01387	P. Fratamico	+	−	+
	O111:NM	00-4748	P. Fratamico	+	+	+
	O111:NM	98-8338	P. Fratamico	+	−	+
	O111:NM	94-0961	P. Fratamico	−	+	+
	O111:NM	96-3166	P. Fratamico	+	+	+
O121	O121:H19	08023	P. Fratamico	−	+	+
	O121:H19	SJ18	P. Fratamico	+	+	+
	O121:H19	03-2832	P. Fratamico	−	+	+
	O121:H19	97-3068	P. Fratamico	−	+	+
O145	O145:NM	SJ23	P. Fratamico	+	+	+
	O145:H28	07865	P. Fratamico	−	+	+
	O145:NM	03-4699	P. Fratamico	+	−	+
	O145:NM	94-0941	P. Fratamico	+	+	+
	O145:NM	83-75	P. Fratamico	−	+	+

a Source is as follows: P. Fratamico, Eastern Regional Research Center, Agricultural Research Center, Agriculture Research Service, U.S. Department of Agriculture, Wyndmoor, Pennsylvania;

b NT: not tested

**Table 2:** Analysis of positive predictive value (PPV) for each serovar grown on different selective media^a^.

*E. coli* serovars	SMAC	CHROMagar	R&F	Rainbow	BHI
**O103**	0.91	NG	0.81	0.92	0.96
**O111**	0.93	0.88	0.97	0.93	0.98
**O121**	0.97	0.94	0.91	0.93	0.94
**O145**	0.91	0.86	0.93	0.89	0.93
**O157**	1	0.95	0.84	0.98	0.94
**O26**	0.95	0.92	0.91	0.94	0.89
**O45**	0.91	0.87	0.9	0.97	0.92

a Obtained by calculating the cross-validation matrix and reporting the positive predictive value [55], [56]. Rainbow and SMAC agar showed the best average PPV for seven serovars tested; in particular SMAC showed 100% of classification for O157.

### Effect of growth media on the rate of colony growth and scatter patterns of serovars

**Figure 3:**
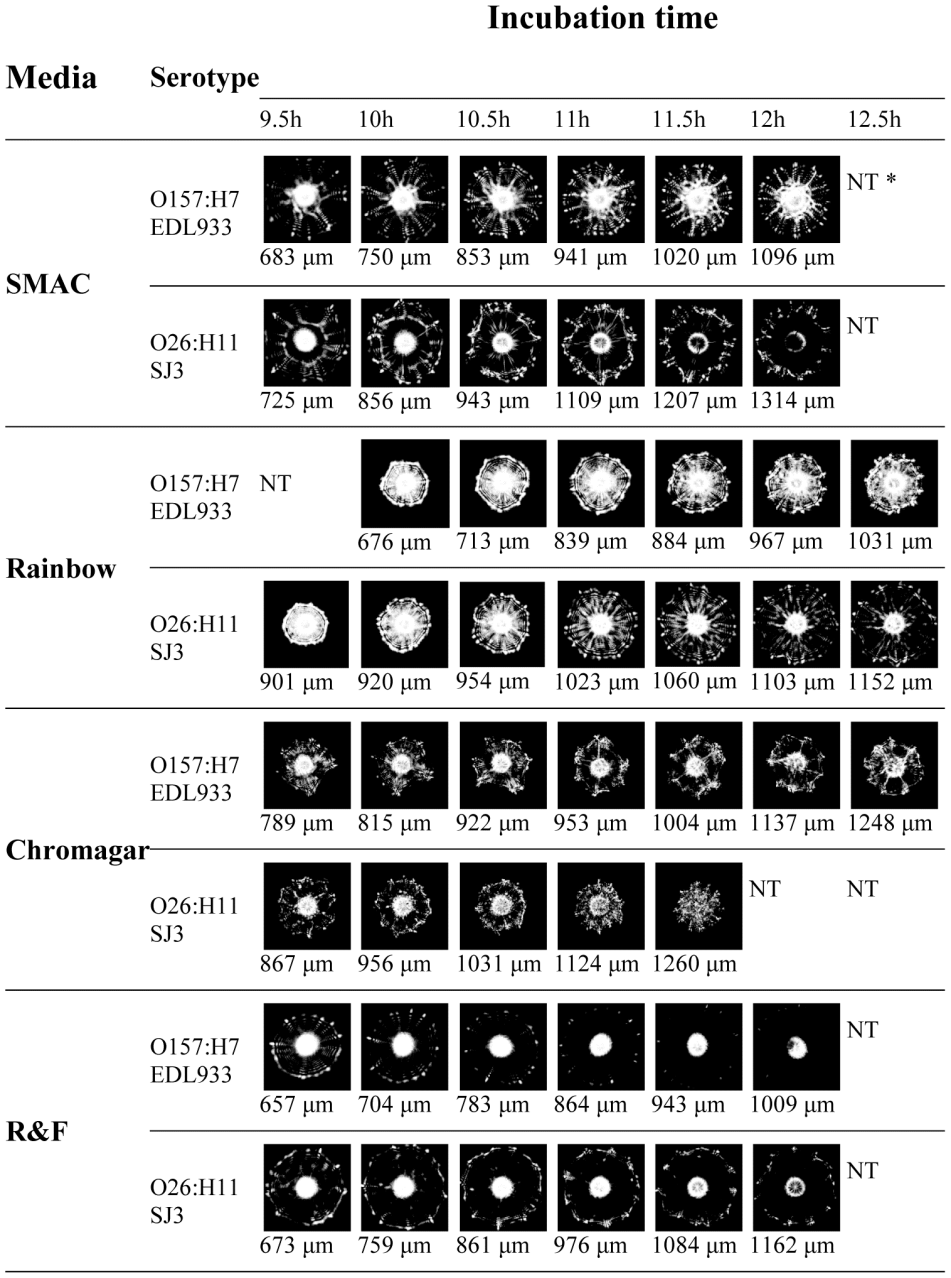
Effect of growth time on light-scatter images of representative O157 and non-O157 STEC strains. Colony sizes (µm) were measured within 5 min before respective scatter patterns were captured by BARDOT. *NT: not tested because no distinct scatter image could be obtained due to oversized colony (>1.2 mm).

Next, we examined colony growth and the changes in scatter patterns over time using the different selective media (**Fig 3**). Previous research suggested that bacterial growth kinetics (expressed as colony diameter) and resultant scatter patterns varied depending on media and strains tested [40], [43]. To determine the correlation between colony growth on SMAC, Rainbow, CHROMagar, and R&F, the corresponding colony scatter patterns of all serovars were examined by BARDOT every 30 min from 9 h to 14 h of incubation at 37°C (**Fig 3** **, Fig S1-S4)**. By close examination of two strains of serogroups, O157 and O26 (**Fig 3**), it was determined that the growth kinetics were generally similar on each growth medium (colony sizes reached 1.0±0.3 mm at 10–12 h of plate incubation); however, a slightly higher growth rate was achieved on SMAC. The optimum colony size selected for scatter experiments with SMAC (**Fig S1**), Rainbow (**Fig S2**), and CHROMagar (**Fig S3**) media were 1.1±0.1 mm, while colonies with smaller sizes (0.8±0.1 mm) were used with R&F medium (**Fig S4**). The smaller colony size for R&F was chosen because of the time-dependent accumulation of black pigment which blocked the interrogating laser in larger colonies (>12 h). In general, the colonies which differed significantly from the optimum size were not selected for BARDOT analysis. The smaller colonies typically produced bright and less distinguishable patterns, while larger colonies generated images with more differential features but of low intensity.

While all representative strains exhibited consistently similar growth morphology on SMAC and Rainbow agar, a higher variability in growth rate among serogroups was observed on CHROMagar and R&F media. Therefore, we established an experimental protocol that employed size-normalized scatter patterns instead of growth-time normalized patterns. The desirable size of the colonies for most serogroups was achieved within 10–12 h of incubation at 37°C, which provided results quickly.

**Figure 4:**
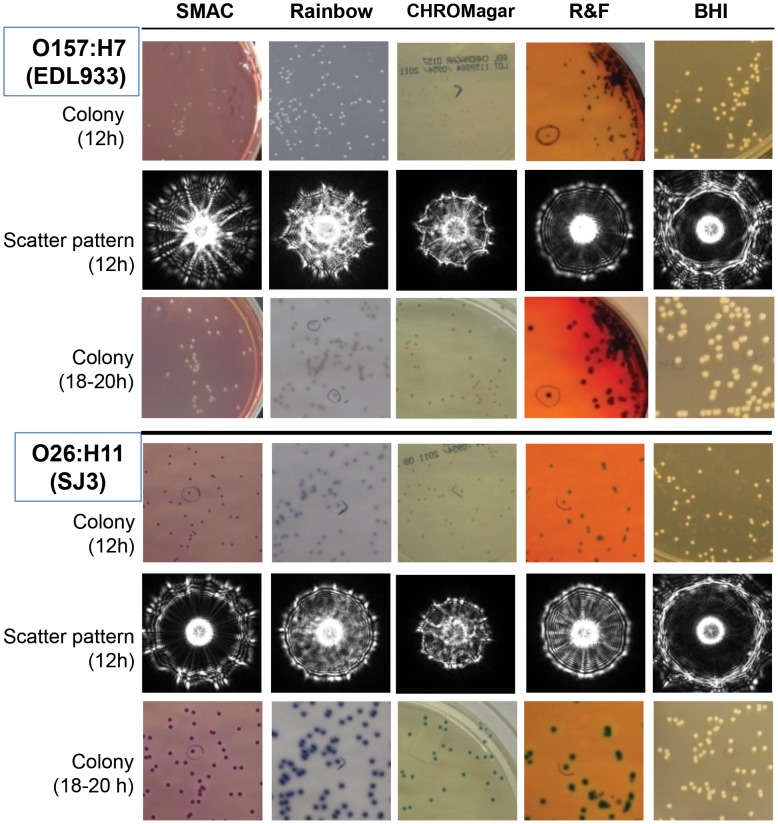
Chromogenic pigment production and scatter patterns of colonies of representative O157 and O26 STEC strains. Plate images and scatter patterns were captured at 10–12 h of incubation when colonies reached approximately 1 mm in diameter. Colony appearance after 18–20 h of growth was also recorded.

When the same bacterial cultures were allowed to continue to grow on the chromogenic media, the colony sizes increased, and color was produced after 18–24 h, aiding in presumptive visual identification (**Fig 4**). The O157 colonies on SMAC agar appeared as dome shaped and colorless, while non-O157 strains produced rose to pink colonies (**Fig 4** **, Fig S5**). On R&F agar, O157 colonies appeared dome shaped with a black precipitate, while non-O157 strains appeared green; however, the color difference between O157 and non-O157 colonies could not be easily distinguished when viewed against a white background (**Fig 4** **, Fig S5**). On Rainbow and CHROMagar, O157 and O111 strains produced grey to black colonies while O26, O45, O103, O121 and O145 produced pink-magenta-purple colonies after 18 h of incubation. On CHROMagar, O157:H7 colonies (mauve-colored) could be distinguished from non-O157 STEC colonies (O111: grey-green; O26, O121 and O145: aqua); however, the colors among non-O157 STEC strains may be too close to be differentiated from each other when multiple strains are present on the same plate. Moreover, colonies on CHROMagar appeared flat with swarming properties. Thus, for all future experiments, SMAC agar was used as the best medium to acquire scatter images of STEC colonies (diameter 1.1±0.1 mm) after 10–12 h of incubation at 37°C.

### Scatter image library

**Figure 5:**
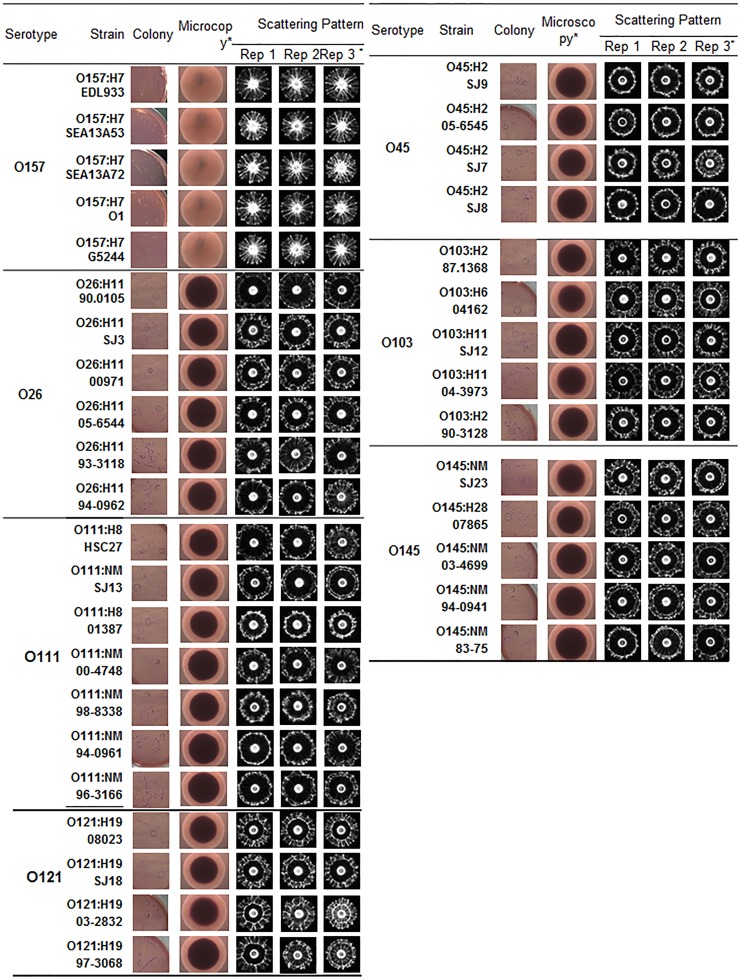
Microscopic colony appearance and scatter images of colonies of STEC serogroups on sorbitol MacConkey (SMAC) agar after 10–12 h of incubation at 37°C. Three representative images for the same strains from three separate experiments are presented. *Rep 3 represents scatter images of colonies presented in this figure.

As indicated above, SMAC produced the best differential scatter signatures among the seven serogroups tested. Analysis of 37 strains representing all seven serogroups (4–7 strains/serogroup) generated highly reproducible scatter patterns for strains within a serogroup (**Fig 5**). Additionally, scatter patterns of 36 O157:H7 (**Fig S6**) and 11 non-O157 strains (**Fig S7**) were also generated on SMAC. Scatter images of other bacteria including *Acinetobacter baumannii*, *Citrobacter freundii*, *Klebsiella pneumoniae*, *Pseudomonas aeruginosa* and *Shigella flexneri* that grew on SMAC were analyzed (**Fig S7**). Scatter patterns of seven serogroups of STEC strains appeared significantly different from those of the non- *E. coli* colonies on SMAC (**Fig S7**). The scatter image library consisted of about 83 strains representing all seven serogroups yielding about 3600–7000 scatter images. These images were stored in image library for use during food sample testing or mixed culture analysis. In addition, scatter image libraries for all seven serovars were also developed after growth on BHI (**Fig S8**), CHROMagar (**Fig S9**), Rainbow (**Fig S10**), and R&F (**Fig S11**) to aid in comparative analysis of any ambiguous serovars.

### Mixed culture testing

**Figure 6:**
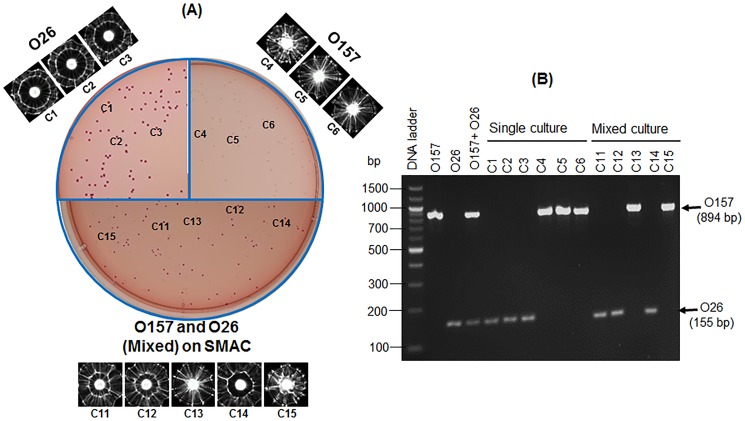
Light-scattering analysis and PCR confirmation of colonies of serogroup O157:H7 (EDL933) and O26:H11 (90-0105) on SMAC agar. (**A**) Scatter images of colonies of O26, O157 and the mixture of O26 and O157 grown on SMAC at 37°C for about 11 h. Representative colonies that were analyzed by multiplex PCR (mPCR) are labeled as C1-C15. (**B**). Agarose gels showing PCR amplified bands for each colony tested.

To further investigate the ability of the light-scattering sensor to detect select *E. coli* serovars from a mixed culture on SMAC agar, two representative serovars (O157 and O26) were co-inoculated on the test medium and the resultant colonies at optimum sizes were analyzed by the light-scattering sensor and confirmed by multiplex PCR (mPCR) [28], [44]. As expected, O157 and O26 generated unambiguous characteristic scatter patterns on SMAC when used singly or in a mixture (**Fig 6**). A typical O157 scatter pattern consisted of radial spokes extending from center to the edge, while the O26 scatter pattern consisted of two outer rings with radial spokes originating from the inner ring. These images were also verified by the image library database. The colony identity of respective serovars was also confirmed by serovar-specific mPCR (**Fig 6B**), where O157 and O26 produced amplified bands of 894 and 155 bp, respectively.

### Food sample testing

**Figure 7:**
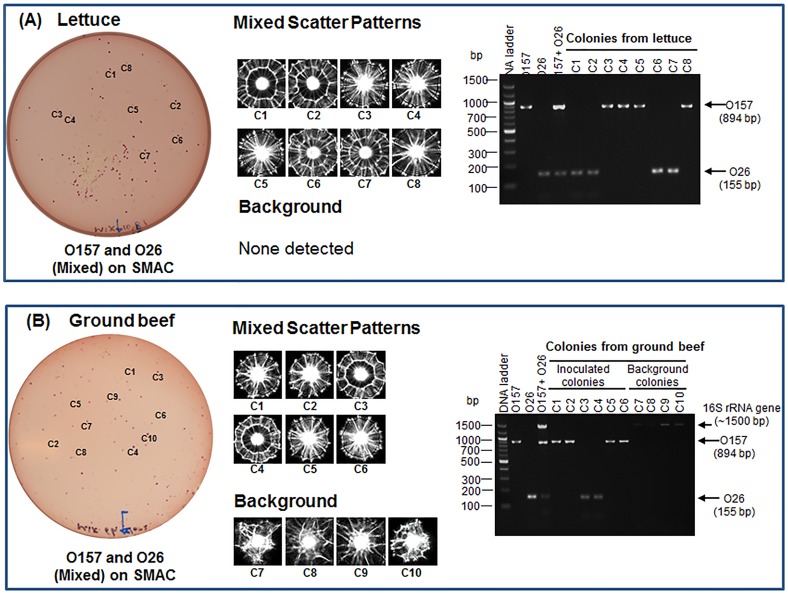
Application of light-scattering sensor for detection and identification of STEC O26 and O157 from inoculated (A) lettuce and (B) ground beef samples. Colonies (c1, c2…) on SMAC are first scanned with BARDOT and then analyzed by mPCR for confirmation. BARDOT accurately detected two serogroups from both food samples and confirmed by PCR. Only ground beef sample contained background non-STEC bacteria whose patterns are different from the STEC serogroups. Moreover, these colonies did not give any amplified products with serovar specific primers but amplified 16S rRNA gene.

Twenty-five grams of each lettuce and ground beef sample were inoculated with mixed cultures of O157 and O26 (50–100 CFU/25 g), enriched in modified tryptic soy broth (mTSB) with novobiocin [42] at 42°C for 10 h, plated on SMAC (at 37°C for 10.5 h), and the colonies were analyzed by BARDOT. The food inoculation experiment was repeated three times with different batches of food. Both O157 and O26 colonies on SMAC were visually indistinguishable after 10.5 h of growth; however, they produced typical scatter patterns when analyzed by BARDOT and were detected unequivocally from inoculated lettuce and ground beef samples (**Fig 7**). Representative colonies of each serovar were also picked from the plates and verified by mPCR, and the identification accuracy was estimated to be 96.6–100% (**Fig 7** **,** **Table 3**). The identification accuracy was defined as the ratio of the number of colonies positively identified with BARDOT and mPCR to the total number of colonies tested.

**Table 3:** Differentiation of mixed cultures of *E. coli* serovars from food samples on SMAC agar based on scatter pattern.

Bacterial/Food sample^a^	No. of colony analyzed	No. of positive colony with BARDOT Library^b^		No. of positive colony with serovars-specific PCR^c^		Identification accuracy (%)^d^
		O26	O157	O26	O157	
*E. coli* O26 & O157 tested as mixed culture	30	15/30	15/30	15/30	14/30^e^	96.6
Lettuce with *E. coli* O26	6	6/6	0/6	6/6	0/6	100.0
Lettuce with *E. coli* O157	6	0/6	6/6	0/6	6/6	100.0
Lettuce with *E. coli* O26 & O157	12	6/12	6/12	5/12	7/12	96.6
Ground beef with *E. coli* O26 & O157	14	7/14	7/14	7/14	7/14	100.0

a Lettuce and ground beef samples were inoculated with 10^2^CFU/25g of each *E. coli* O26 and *E. coli* O157 cells

b *E. coli* colonies grown on SMAC agar for 10–11 h were matched with BARDOT library containing multiple standard strains of O157 STEC and non-O157 STEC.

c *E. coli* serovar (O26 and O157) specific primers were used in multiplex PCR (mPCR). Prior to food sample analysis primers were validated for specificity with three standard strains of each *E. coli* O157:H7 and O26:H11. Primer sequences were adapted from reference [28].

d Identification accuracy measures the ratio of number of colonies correctly identified with BARDOT as well as PCR and total number of colonies analyzed. Identification of colonies with BARDOT and mPCR were performed as a blind study.

e One colony (#R1-3/C1) analyzed with mPCR did not produce any amplified products, however, it was identified as O157 serovar after analysis with BARDOT library.

With lettuce testing, there were no non-O157/non-O26 background colonies on SMAC, but several background colonies were obtained from ground beef (**Fig 7B**). These background colonies also did not amplify either O157 or O26-specific genes. These data clearly show that BARDOT is capable of detecting two STEC serovars in a mixture in the presence of background microbiota in less than 24 h. Of note, none of the uninoculated food samples tested in this study contained any of the seven STEC serovars when used as controls.

## Discussion

This paper is the first demonstration of the application of BARDOT to detect and differentiate colonies of seven STEC serogroups on agar plates based on colony scatter signatures. Initially four selective chromogenic media (SMAC, Rainbow, CHROMagar and R&F) were evaluated and based on the scatter pattern classification accuracy, SMAC provided the best results for STEC detection. Rainbow agar also provided high accuracy in differentiation of serogroups and may also be used for STEC detection using BARDOT. CHROMagar O157, and R&F *E. coli* O157:H7 are designed primarily for isolation of O157:H7 and may not support good growth of non-O157 STEC serovars. Moreover, scatter pattern classification accuracy values for CHROMagar O157, and R&F *E. coli* O157:H7 were slightly lower than SMAC and Rainbow, and thus they may not be suitable for BARDOT-based detection. Even though nonselective BHI agar had a very high accuracy in discriminating the seven STEC serogroups, it may not be suitable for testing of real-world food samples because of potential interference from background natural microbiota; however, BHI agar may be useful for differentiation or verification of pure cultures of STEC serovars. Recently, several new commercial selective and differential chromogenic media (ChromID O157:H7, bioMerieux; Harlequin SMAC-BCIG, Lab M; HardyCHROM E. coli O157; CHROMagar STEC, CHROMagar Microbiology [45]) have been marketed by many vendors, which could be tried with BARDOT. However, it is important to ascertain that there is no interference of the agar medium with laser propagation, since the BARDOT system generates forward scatter patterns of colonies.

We have indicated earlier that classification accuracy of colonies of different species from the same genera [37], [40] or serovars from a single species [41] varies depending on the growth medium used, and this suggests that bacterial colony morphology and composition can be leveraged for accurate classification. This is possible due to the photon-cell interaction, which results in unique and reproducible scatter patterns [37], [39]. The colony scatter pattern is a phenomenon that occurs due to the accumulation of the interactions of incoming photons across the depth of the colony. Their physical (color, size, shape, roughness, and thickness) or chemical (metabolic by-product composition) characteristics modify the optical amplitude and phase, which scatters into an imaging plane to create a scatterogram [43], [46], [47].

In addition to colony size, the height of colonies is another important physical determinant for differential scatter patterns [37], [43]. We observed that elevated colonies on SMAC and Rainbow agar formed more diffused patterns, while colonies on CHROMagar appeared flat, and thus produced relatively more concentrated patterns with less distinctive ring structures (**Fig 2**), yielding a lower classification accuracy.

Chromogenic selective or differential media are integral components of classical microbiological methods for presumptive isolation of pathogens from test samples. The chromogens are produced as a result of bacterial fermentative and enzymatic activities [48], [49], and thus it is speculated that differences in scatter patterns observed for different serovars are affected by the accumulation of bacterial metabolic by-products or chromogenic components [38], [39]. Furthermore, the O-antigen in lipopolysaccharides (LPS) has been suggested as a possible contributing factor for colony morphology that can be interrogated by the light-scattering technology [32]–[34] and possibly an important discriminant for differential scatter patterns. LPS is a major component of the outer membrane of Gram-negative bacteria, and it is composed of lipid A, core oligosaccharides, and O-polysaccharides (or O-antigen). The O-(somatic) antigen is attached to the outermost domain of core oligosaccharide, while lipid A anchors to the inner core. While lipid A and core oligosaccharide are highly conserved or have limited variations, the compositional structure of O-antigens varies considerably among serogroups of pathogenic *E. coli* [50]–[52] and even the seven serovars of STEC [28], [53] studied here. Although the size of the O-antigen is quite small (outer membrane is ∼40 nm) compared to the interrogating laser wavelength used here (635 nm), the collective appearance and characteristics of the O-antigen at the surface of bacterium in a colony can provide differential scatter patterns. The H (flagellar) antigen did not appear to play any role, since scatter patterns of nonflagellated/non motile O111:NM or O145:NM strains were very similar to flagellated strains of O111:H8 or O145:H18 strains (**Fig 5**).

BARDOT was successful in detecting and differentiating colonies of serogroups O157 and O26 from mixed cultures and even from inoculated food systems in the presence of background microbiota. BARDOT detected these two pathogens in less than 24 h (10 h of enrichment in mTSB plus 10.5 h of on-plate incubation) in lettuce and ground beef inoculated with low levels (50–100 CFU/25g) of O157 and O26,suggesting its potential for screening of food samples for STEC. BARDOT-positive colonies were further confirmed by PCR. Since BARDOT is a noninvasive and non-destructive technique, colonies can be used for further molecular or pathophysiological characterization, if necessary.

In this study, we tested seven major STEC serovars (about 73 strains) and 10 additional miscellaneous serovars. BARDOT showed high accuracy in differentiation among the seven major STEC serovars. Continued testing of additional serovars needs to be done in order to determine any overlapping patterns among common serovars and to create a robust *E. coli* library on SMAC, which is currently in progress. The growth rate of serovars also varies depending on the strain and the medium used, which makes it difficult to identify the optimal, fit-for-all detection time for BARDOT-based detection. A fully automated system equipped with an incubator, plate handling robot, and the laser scanner would be useful. Such an automated system is currently under development by Advanced Bioimaging Systems (advancedbioimgaingsystems.com), which may be used to overcome this technical obstacle.

## Conclusion

Our results demonstrate that BARDOT can detect and discriminate the colonies of seven major STEC serovars with high accuracy. Among the media tested, SMAC and Rainbow provided the best results allowing serogroup differentiation with over 90% accuracy at an early stage of colony growth when colony morphology and color are indistinguishable. BARDOT-based detection can be completed in less than 24 h, which includes sample enrichment in mTSB for 10 h followed by growth on SMAC for 10–12 h, allowing users to have access to the isolated colonies for further characterization. BARDOT could potentially be used as a real-time screening tool for on-plate differentiation of the most important STEC serogroups as they grow on selective agar plates.

## Materials and Methods

### Bacterial strains and culture conditions

A total of thirty-seven *E. coli* isolates belonging to serogroups O157, O26, O45, O103, O111, O121, and O145 were tested in this study (**Table 1**). In addition, 36 strains of O157:H7 (**Fig S6**), 11 miscellaneous *E. coli* strains and five other bacterial cultures including *Acinetobacter baumannii* NRRL B41237, *Citrobacter freundii* NRRL B2643, *Klebsiella pneumoniae* NRRL B41958, *Pseudomonas aeruginosa* ATCC 10145 and *Shigella flexneri* PRI 387 that grew on SMAC(**Fig S7**) were used for scatter image library development. Working cultures were prepared by inoculating a loop full of frozen bacterial stocks (−80°C) into 3 ml BHI broth and incubating at 37°C for 12–15 h. Cultures were serially (10-fold) diluted in phosphate buffered saline (PBS; 1.38g/L of sodium phosphate monobasic, 2.68g/L of sodium phosphate dibasic, 8.5g/L of 0.85% sodium chloride) prior to plating appropriate dilutions on chromogenic media to obtain a colony population of 30–100 colonies per plate.

### Media and phase contrast microscopy

Four types of differential chromogenic media were used to grow bacterial cultures for light-scattering experiments. The media included: BHI agar, Sorbitol MacConkey (SMAC; Becton Dickinson) agar, Rainbow Agar O157 (Biolog, Hayward, CA), BBL CHROMagar O157 (Becton Dickinson), and R&F *E. coli* O157:H7 medium (R&F Products, Downers Grove, IL). Agar plates were prepared as instructed by the manufacturer, tempered to 45°C, dispensed 20 mL/plate, cooled at room temperature for 10–20 min, and stored in a sealed plastic bag until use (used within 7–30 days) [54]. After plating appropriate dilutions of bacterial cultures, the plates were incubated at 37°C for 10–12 h or until the colony size reached 1.1±0.1 mm in diameter. The colony size and morphology of bacterial cultures were examined under a 10× PH1 objective of a Leica DMLB microscope (Leica Microsystems USA, Bannockburn, IL) equipped with a SPOT RT color camera (Diagnostic Instruments, Inc). Images were captured using SPOT Advanced software 4.6.4.2 (Diagnostic Instruments, Inc).

### Light-scattering instrumentation and image acquisition

A prototype light scattering sensor known as the BARDOT system (Advanced Bioimaging Systems, West Lafayette, IN) consisting of two main functionalities was used. **Figure 1** shows instrumentation setup and diagrammatic representation of the operating principle. In brief, the standard Petri dish with the grown bacterial colony is imaged to obtain a map showing location of each colony, where the centroid locations of each colony are automatically identified. The cut-off value for absorption and circularity was set to filter the non-bacterial or doublet colonies while the colony center locations were transferred to perform the forward scattering measurement automatically. BARDOT moves the Petri dish to the respective center of the colony such that it aligns with the incoming 635 nm laser which generates the forward scattering patterns. Detailed hardware and software development and specifications were published earlier [38], [39]. To build scatter image libraries, colony scatter patterns of 50–100 colonies per strain of each serogroup were collected.

### Image analysis

The process of scatter-pattern analysis has been described in detail in our previous reports [55], [56]. Briefly, for each individual scatter pattern characterizing a single *E. coli* colony, a total of 78 features are extracted. The quantitative pattern characteristics include 65 pseudo-Zernike orthogonal moments and 13 Haralick texture features. The features were concatenated, forming feature vectors for each colony. We used either the entire feature set (for the 7-serovar library), or the most informative features selected by a random-forest technique [57], [58]. The extracted features were used to find the best combination of parameters for a Gaussian kernel-based support vector machine (SVM) classifier. All the tested classifiers were trained and their performance was evaluated using 10-times cross-validation. In every round of cross-validation, the data set was partitioned randomly into training and testing subsets. The classifiers were retrained independently on every training subset, and then the remaining testing subset was classified. The results of all the cross-validation rounds were summarized in a confusion matrix, which was subsequently used to compute sensitivity, specificity, positive predictive value (PPV), and negative predictive value (NPV) [59]. The sensitivity describes the probability that our classifier will produce a true result when used on a population of colonies also containing colonies representing a serotype of interest. The specificity illustrated the probability that the test will produce a true negative result when used on colonies formed by organisms other than the serotype of interest. The PPV gives the probability that a colony truly belongs to a serotype of interest when a BARDOT system claims so. The NPV is the probability that a colony does not represent a serotype of interest when a negative result is returned.

### Mixed culture testing

One representative STEC strain belonging to serogroups O26 and O157 (*E. coli* O26:H11 90-0105 and *E. coli* O157:H7 EDL 933) were selected to prepare two-strain cocktails. Each bacterial culture was grown individually in 3 ml of BHI broth for overnight, and then equal amounts (1 ml) of each were mixed. The mixed cultures of O157 and O26 were serially diluted to the appropriate dilutions and surface-plated onto SMAC agar plates and incubated at 37°C until the colony grew to optimum sizes as described earlier. The scatter images of individual colonies were captured by the BARDOT system, and five colonies were selected for PCR confirmation after matching with the *E. coli* serovar library generated on SMAC medium.

### Food sample testing

Romaine lettuce (triple washed) and ground beef samples were purchased from local grocery stores (West Lafayette, IN) on the day of the experiment. A strain of serovar O26 and O157 (O26:H11 90-0105, and O157:H7 EDL933) was used for artificial inoculation of food samples. Overnight grown cultures were diluted to obtain 5×10^2^–1×10^3^ CFU/mL. The cell number in the inoculum was determined by colony counts after making serial dilutions, plating onto SMAC, and incubation at 37°C for 18 h. A 100 µl-aliquot of the diluted cultures was inoculated onto a lettuce sample (25 g) to obtain inoculum levels of 50–100 CFU/25g. Inoculated samples were air-dried in a class II biosafety cabinet with a constant laminar flow at 22±2°C for 15 min to allow attachment of bacteria to leaf surfaces. After air-drying, lettuce samples were aseptically transferred into Seward filter stomacher bags (Model 400 Bags, Seward Ltd, West Sussex, UK) containing 225 ml of modified TSB (mTSB) plus 8 mg/L of novobiocin (N-1628, Sigma) [42], [60]. The resulting samples were incubated at 42°C for 10 h with continuous shaking at 130 rpm. As negative controls, uninoculated samples were treated in a similar manner but using sterile PBS in place of the inoculum. The enriched samples were serially diluted and surface plated onto SMAC agar. All colonies on plates were examined by BARDOT after incubation at 37°C for 10–12 h when colonies reached appropriate sizes. Five randomly picked colonies from each plate were subjected to a multiplex polymerase chain reaction (mPCR) assay for verification of *E. coli* serovars (O157 and O26) used in the experiment.

### Multiplex PCR

The identity of representative *E. coli* colonies scanned by BARDOT was further confirmed for O-antigen specific genes (**Table S1**) using an mPCR assay as described previously [28], [44]. DNA samples from *E. coli* serovars were extracted using the FDA-BAM protocol for DNA template preparation for real-time PCR screening [42]. Proper positive (DNA from standard *E. coli* strains) and negative (deionized water) controls were used for each PCR reaction during validation and testing of PCR primers and colonies from mixed cultures and from food sample enrichments. As a control, amplification of the 16S rRNA gene (**Table S1**) was used to confirm background colonies obtained from ground beef samples.

## Supporting Information

Figure S1 **Forward-scatter images of colonies of representative strains from STEC serogroups O157, O26, O45, O103, O111, O121, and O145 grown on SMAC agar.** Colony sizes are measured by light microscopy immediately before light-scatter screening, and the diameter (µm) of each colony is indicated below respective scatter images.
(TIF)

Figure S2 **Forward-scatter images of colonies of representative strains from STEC serogroups O157, O26, O45, O103, O111, O121, and O145 grown on Rainbow agar.** Colony sizes were measured by light microscopy immediately before light-scatter screening, and the diameter (µm) of each colony is indicated below respective scatter images.
(TIF)

Figure S3 **Forward-scatter images of colonies of representative strains from STEC serogroups O157, O26, O45, O103, O111, O121, and O145 grown on CHROMagar.** Colony sizes were measured by light microscopy immediately before light-scatter screening, and the diameter (µm) of each colony is indicated below respective scatter images. NG, No growth, NT, Not tested
(TIF)

Figure S4 **Forward-scatter images of colonies of representative strains from STEC serogroups O157, O26, O45, O103, O111, O121, and O145 grown on R&F medium agar.** Colony sizes were measured by light microscopy immediately before light-scatter screening, and the diameter (µm) of each colony is indicated below respective scatter images.
(TIF)

Figure S5 **Coloration and scatter patterns of colonies of representative STEC strains from each serogroup.** Scatter patterns were generated at 10–12 h of incubation when colonies reached approximately 1 mm in diameter, while plate images were captured at both 10–12 h and 18–20 h of incubation to demonstrate color change over time. * NG: no growth of O103 strains on CHROMagar. *N/A: images were not captured
(TIF)

Figure S6 **Scatter images of** ***E. coli*** **O157:H7 strains on SMAC agar after 10–12 h of growth.**
(TIF)

Figure S7 **Scatter images of (A)** ***E. coli*** **non-O157 strains and (B) other bacterial cultures including** ***Acinetobacter baumannii*** **,** ***Citrobacter freundii*** **,** ***Klebsiella pneumoniae*** **,** ***Pseudomonas aeruginosa*** **and** ***Shigella flexneri*** **on SMAC agar after 10–12 h of growth.**
(TIF)

Figure S8 **Representative images of colonies on plate, light microscopic images of individual colony and scatter patterns of STEC serovars grown on BHI agar.** All images were collected after about 10.5 h of incubation at 37°C. *Rep 3 represents the scatter patterns of microscopic images of colonies presented in this figure.
(TIF)

Figure S9 **Representative images of colonies on plates, light microscopic images of individual colony and scattering patterns of STEC serovars grown on CHROMagar.** All images were collected after about 10.5 h of incubation at 37°C. *Rep 3 represents the scatter patterns of microscopic images of colonies presented in this figure.
(TIF)

Figure S10 **Representative images of colonies on plates, light microscopic images of individual colony and scatter patterns of STEC serovars grown on Rainbow agar after about 10 h of incubation at 37°C.** Colony images were also captured after 18 h to illustrate visible color change. *Rep 2 represents the scatter patterns of microscopic images of colonies presented in this figure.
(TIF)

Figure S11 **Representative images of colonies on plates, light microscopic images of individual colony and scatter patterns of STEC serovars grown on R&F O157:H7 after about 10 h of incubation at 37°C.** Colony images were also captured after 18 h to illustrate visible color change. *Rep 2 represents the scatter patterns of microscopic images of colonies presented in this figure.
(TIF)

Table S1 **Oligonucleotide primers used for detection of virulence and O-antigen genes of** ***E. coli*** **serovars.**
(DOCX)
