# Supplementary figures and images for: Light Scattering Sensor for Direct Identification of Colonies of Escherichia coli Serogroups O26, O45, O103, O111, O121, O145 and O157
Source: PLoS One. 2014 Aug 19;9(8):e105272. doi: 10.1371/journal.pone.0105272 (PMC4138183; doi:10.1371/journal.pone.0105272)

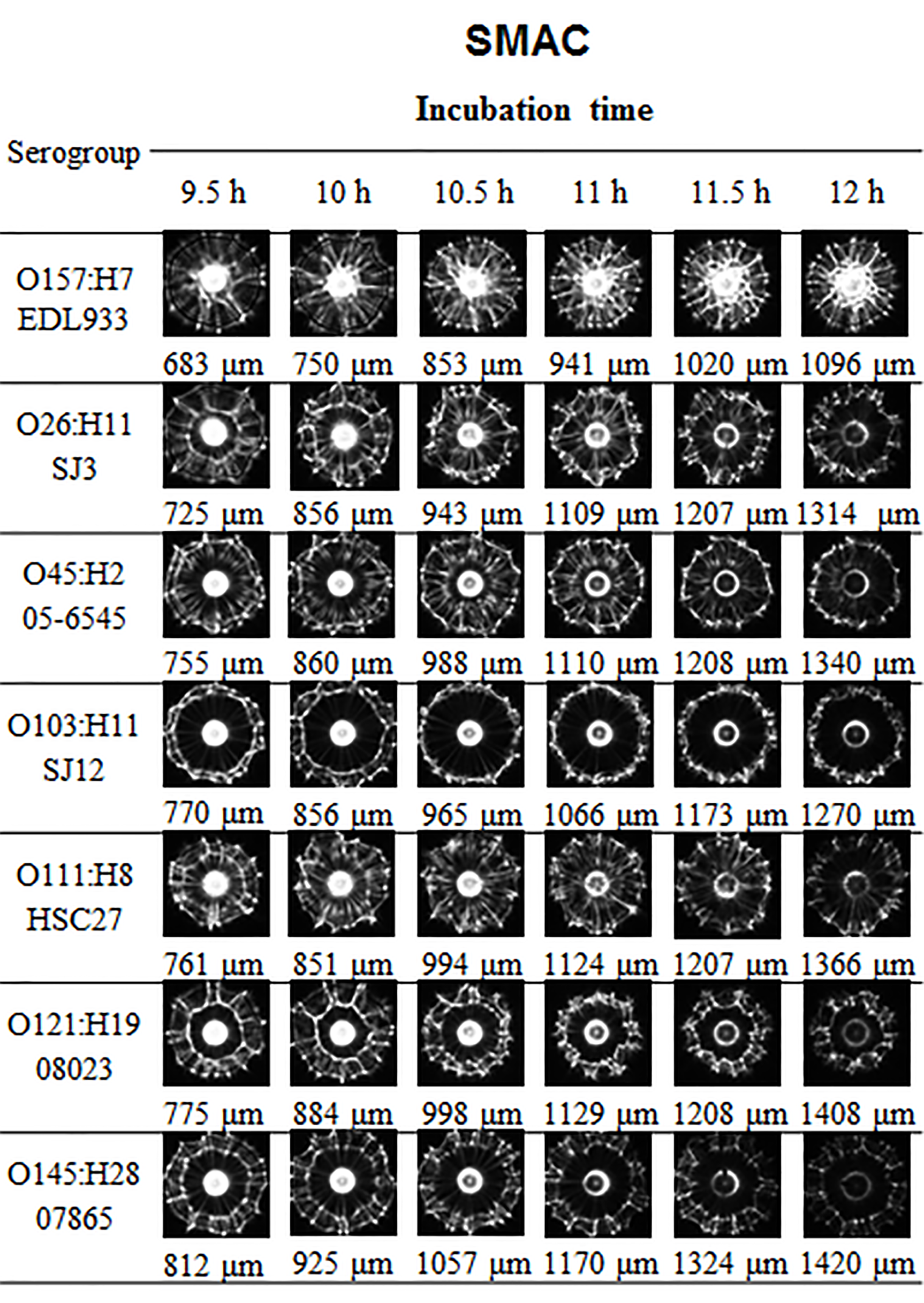

Supplement: Figure S1 — Forward-scatter images of colonies of representative strains from STEC serogroups O157, O26, O45, O103, O111, O121, and O145 grown on SMAC agar. Colony sizes are measured by light microscopy immediately before light-scatter screening, and the diameter (µm) of each colony is indicated below respective scatter images. (TIF) [file pone.0105272.s001.tif]

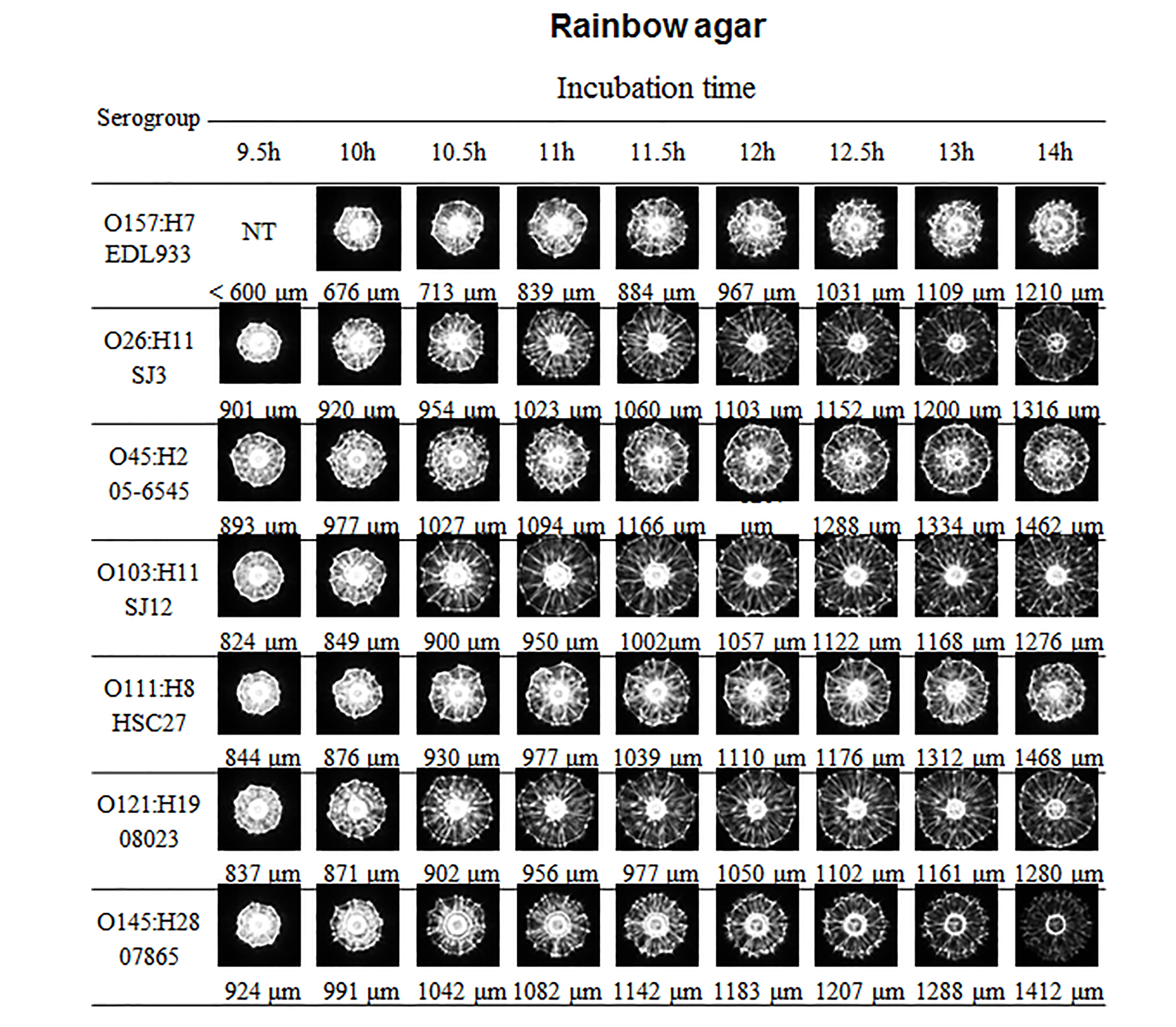

Supplement: Figure S2 — Forward-scatter images of colonies of representative strains from STEC serogroups O157, O26, O45, O103, O111, O121, and O145 grown on Rainbow agar. Colony sizes were measured by light microscopy immediately before light-scatter screening, and the diameter (µm) of each colony is indicated below respective scatter images. (TIF) [file pone.0105272.s002.tif]

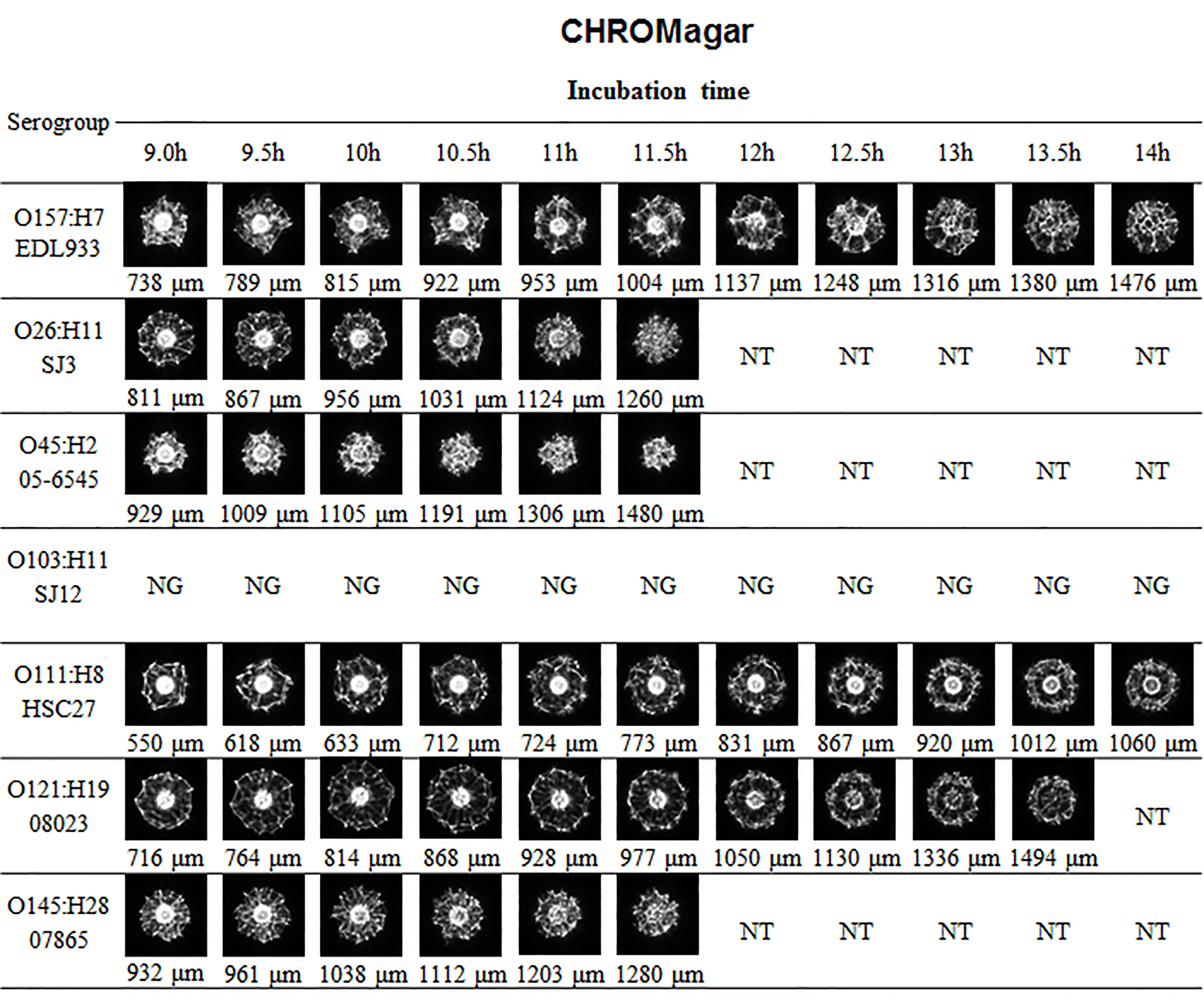

Supplement: Figure S3 — Forward-scatter images of colonies of representative strains from STEC serogroups O157, O26, O45, O103, O111, O121, and O145 grown on CHROMagar. Colony sizes were measured by light microscopy immediately before light-scatter screening, and the diameter (µm) of each colony is indicated below respective scatter images. NG, No growth, NT, Not tested (TIF) [file pone.0105272.s003.tif]

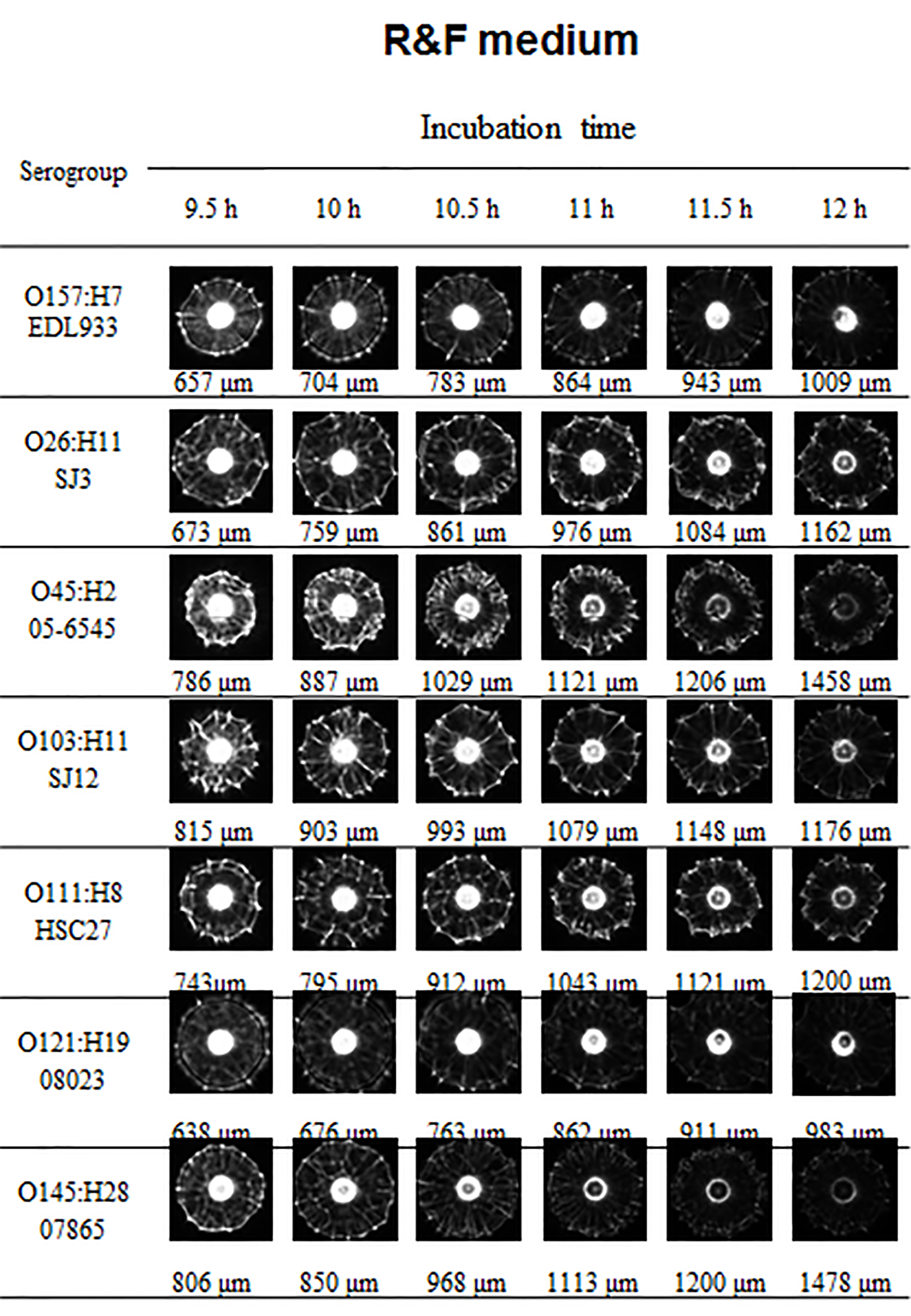

Supplement: Figure S4 — Forward-scatter images of colonies of representative strains from STEC serogroups O157, O26, O45, O103, O111, O121, and O145 grown on R&F medium agar. Colony sizes were measured by light microscopy immediately before light-scatter screening, and the diameter (µm) of each colony is indicated below respective scatter images. (TIF) [file pone.0105272.s004.tif]

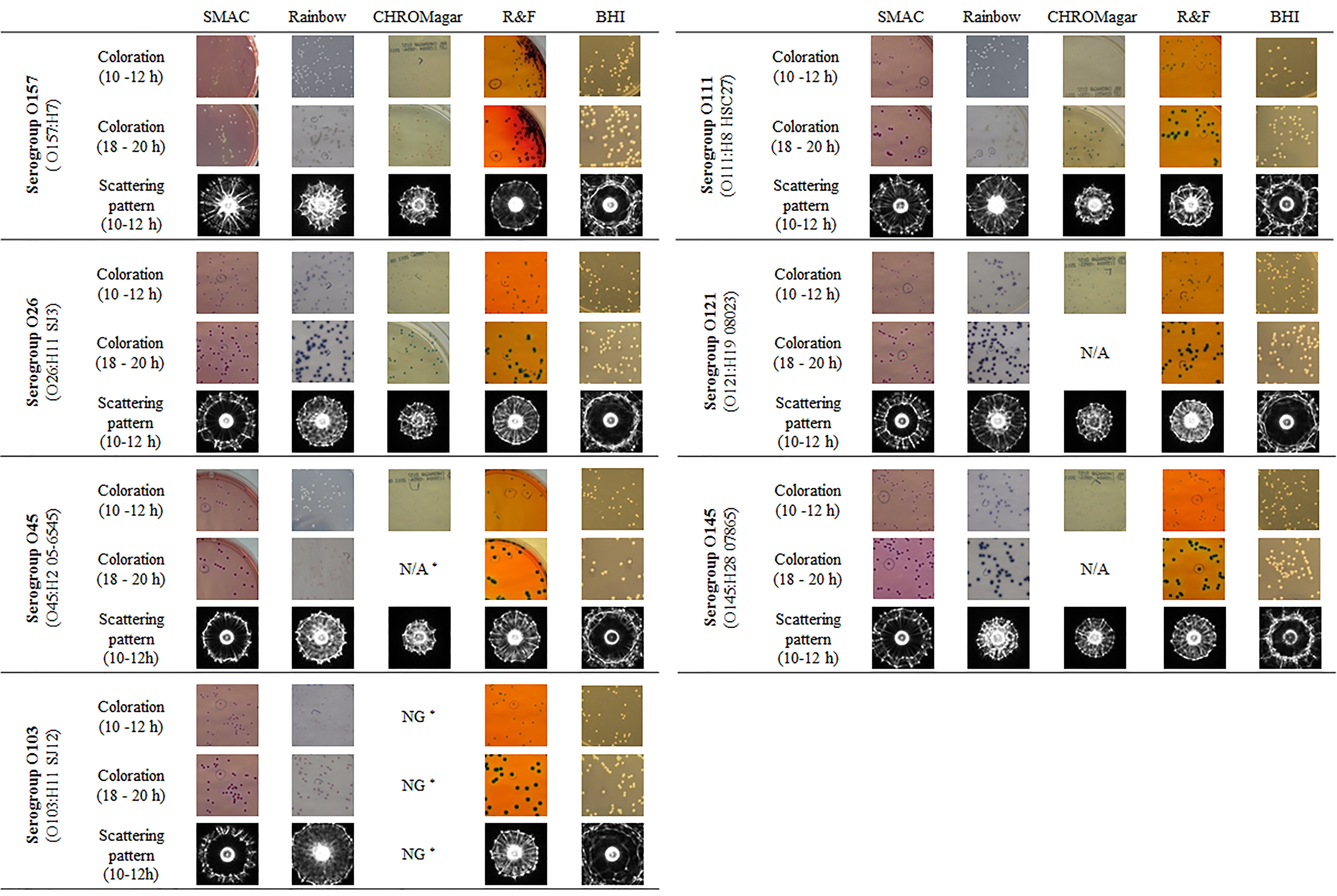

Supplement: Figure S5 — Coloration and scatter patterns of colonies of representative STEC strains from each serogroup. Scatter patterns were generated at 10–12 h of incubation when colonies reached approximately 1 mm in diameter, while plate images were captured at both 10–12 h and 18–20 h of incubation to demonstrate color change over time. * NG: no growth of O103 strains on CHROMagar. *N/A: images were not captured (TIF) [file pone.0105272.s005.tif]

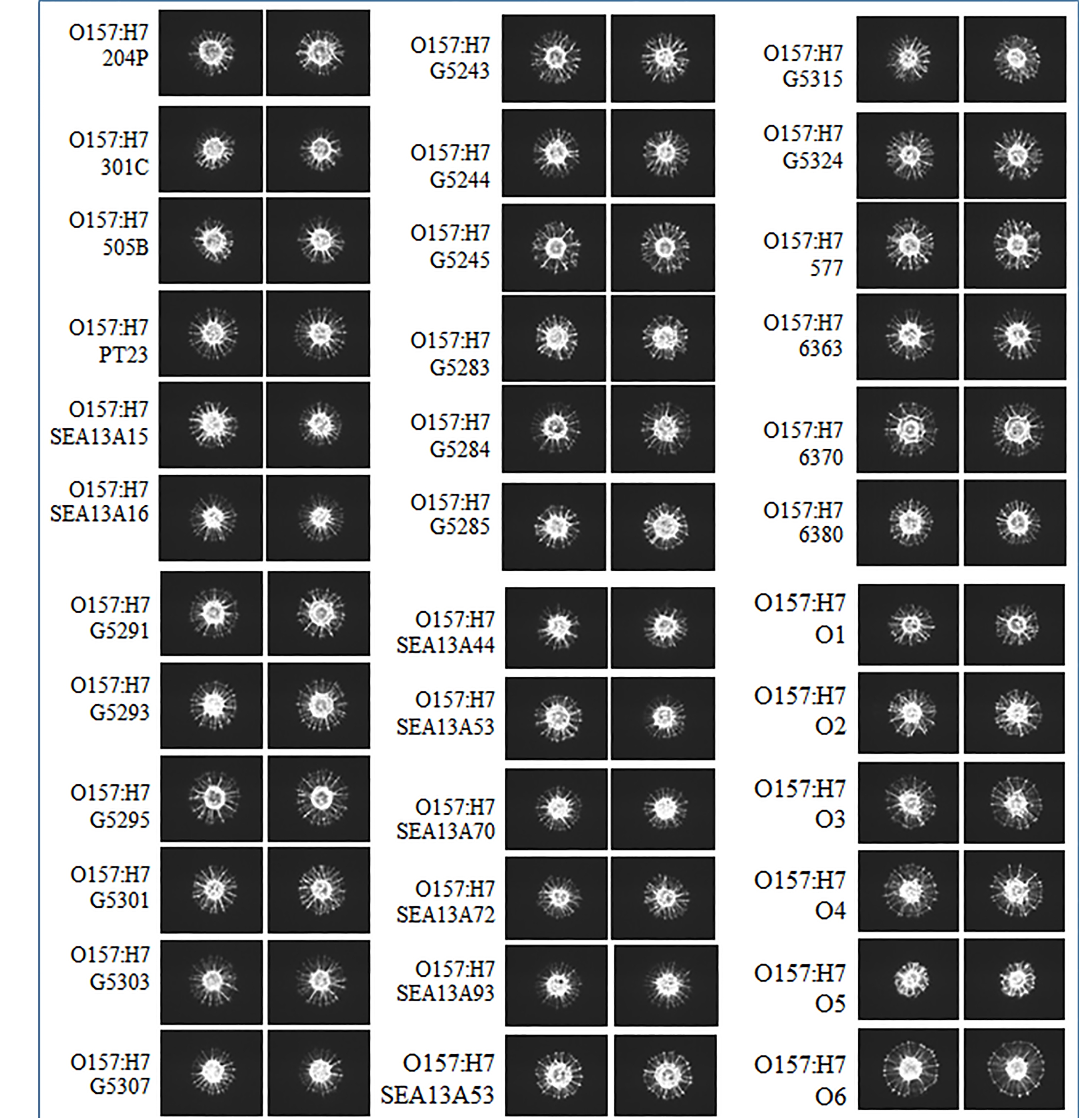

Supplement: Figure S6 — Scatter images of E. coli O157:H7 strains on SMAC agar after 10–12 h of growth. (TIF) [file pone.0105272.s006.tif]

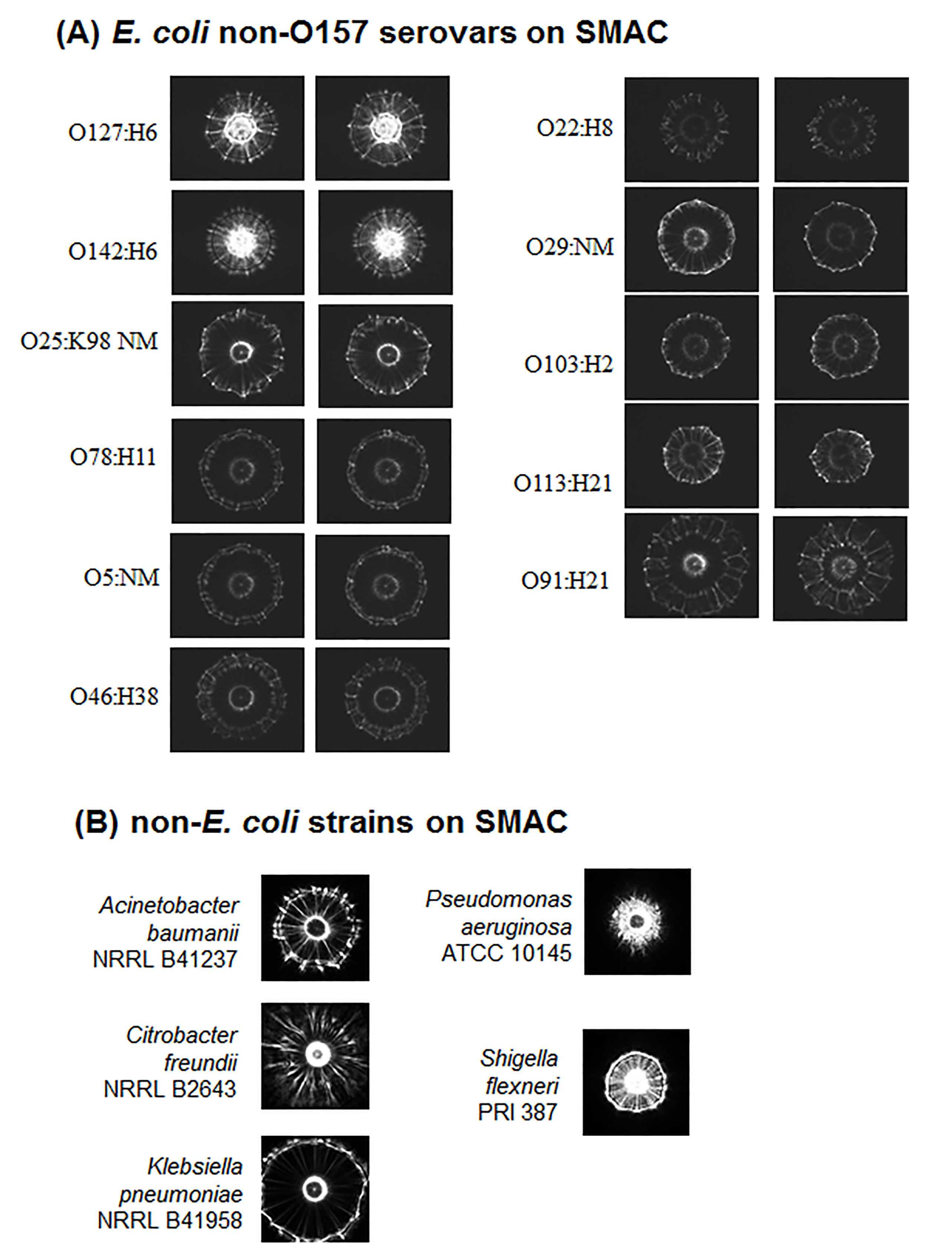

Supplement: Figure S7 — Scatter images of (A) E. coli non-O157 strains and (B) other bacterial cultures including Acinetobacter baumannii , Citrobacter freundii , Klebsiella pneumoniae , Pseudomonas aeruginosa and Shigella flexneri on SMAC agar after 10–12 h of growth. (TIF) [file pone.0105272.s007.tif]

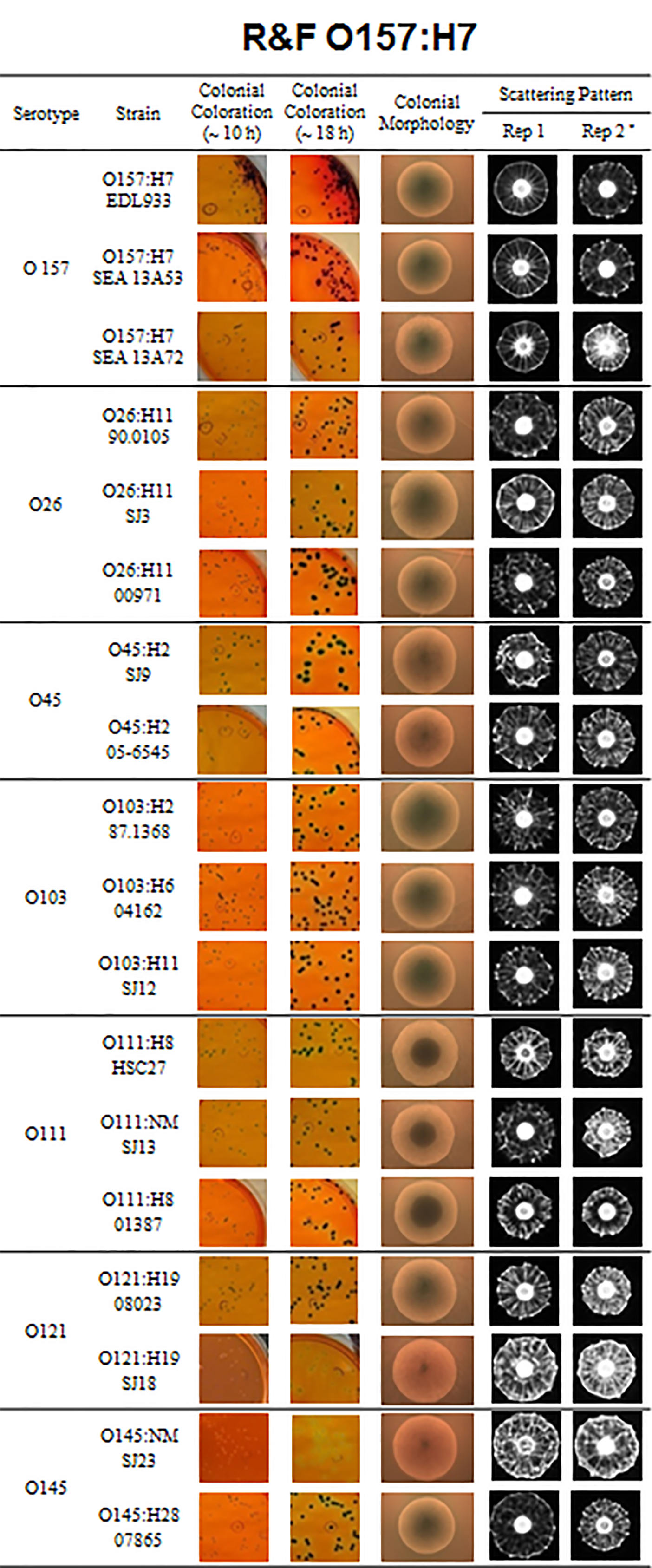

Supplement: Figure S8 — Representative images of colonies on plate, light microscopic images of individual colony and scatter patterns of STEC serovars grown on BHI agar. All images were collected after about 10.5 h of incubation at 37°C. *Rep 3 represents the scatter patterns of microscopic images of colonies presented in this figure. (TIF) [file pone.0105272.s008.tif]

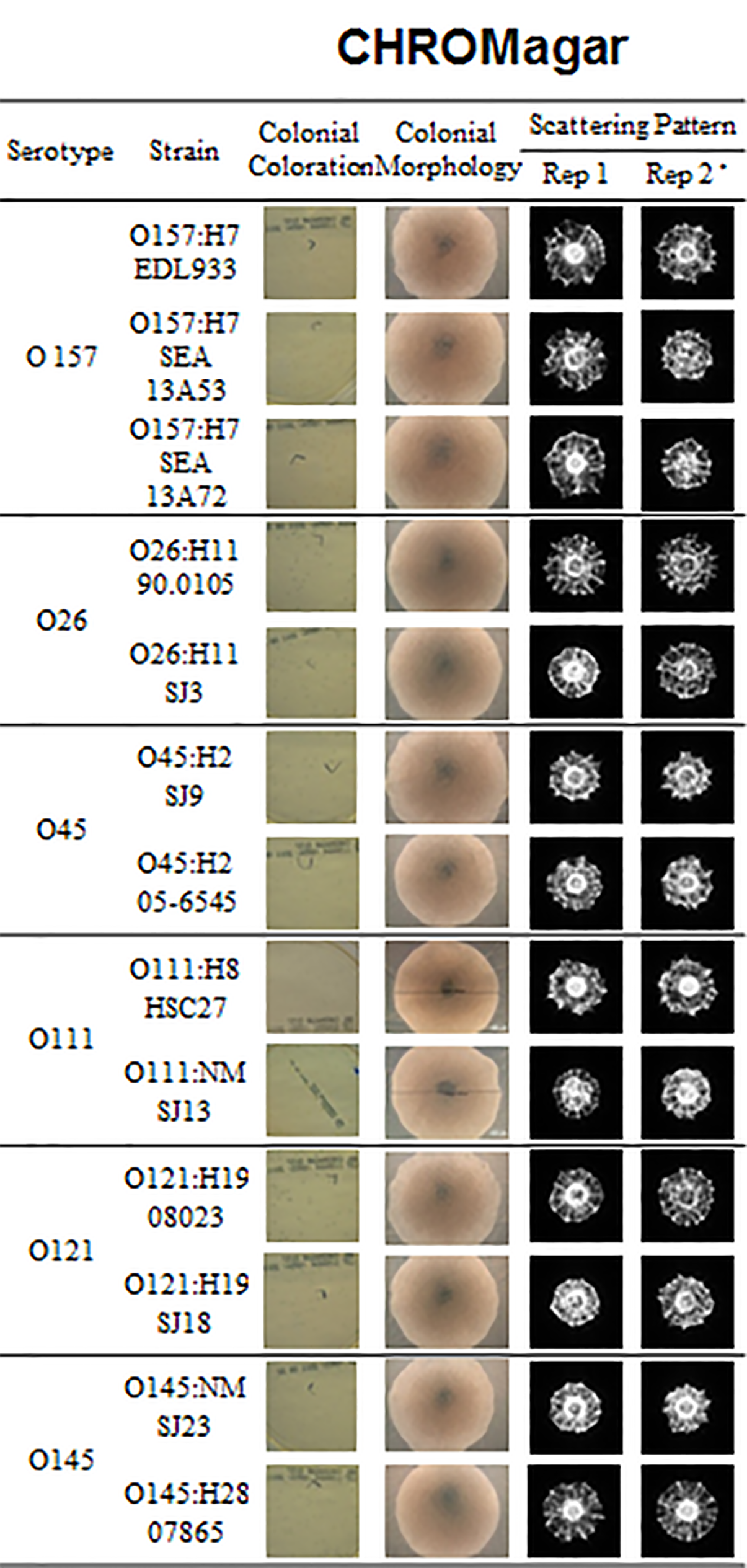

Supplement: Figure S9 — Representative images of colonies on plates, light microscopic images of individual colony and scattering patterns of STEC serovars grown on CHROMagar. All images were collected after about 10.5 h of incubation at 37°C. *Rep 3 represents the scatter patterns of microscopic images of colonies presented in this figure. (TIF) [file pone.0105272.s009.tif]

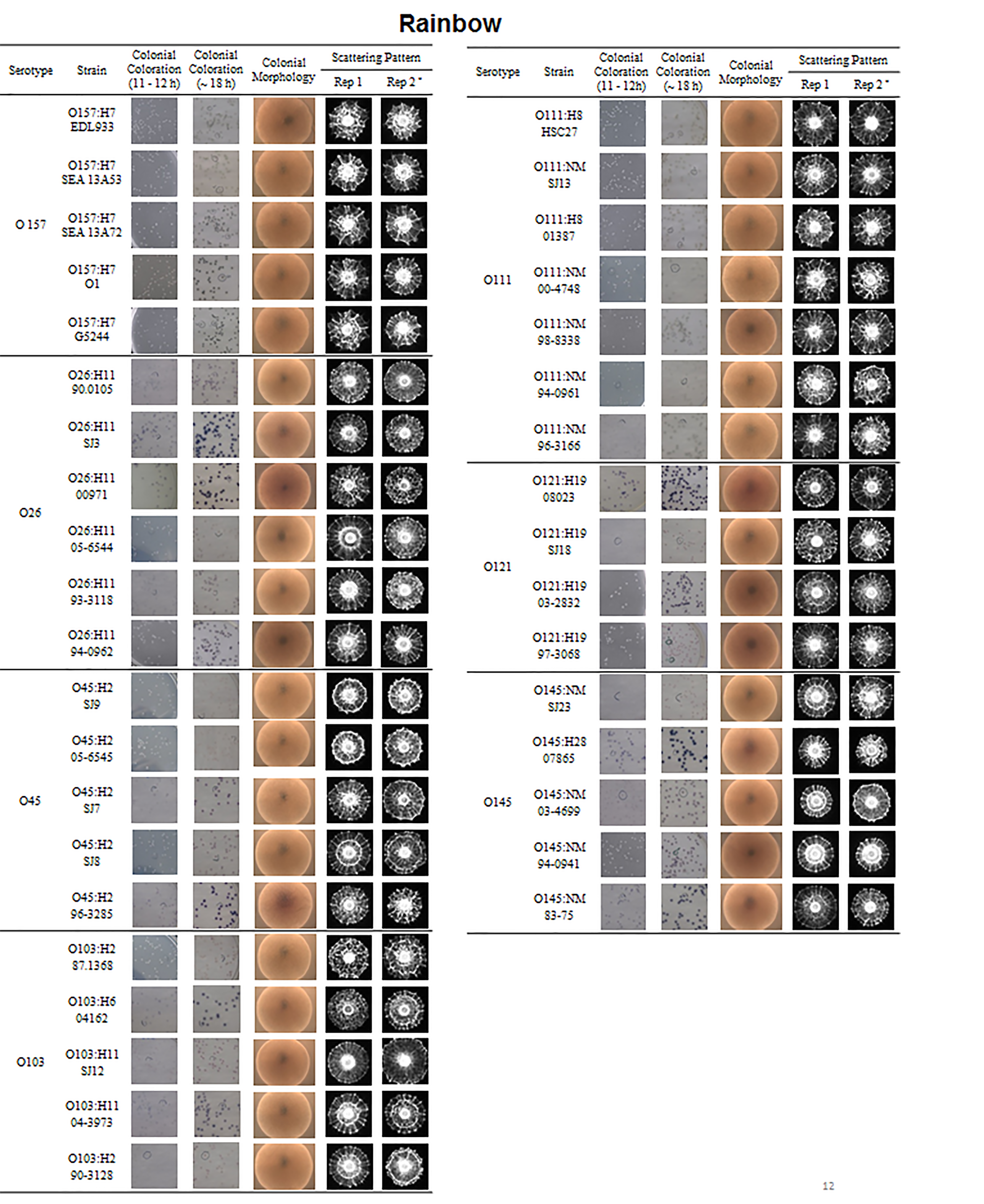

Supplement: Figure S10 — Representative images of colonies on plates, light microscopic images of individual colony and scatter patterns of STEC serovars grown on Rainbow agar after about 10 h of incubation at 37°C. Colony images were also captured after 18 h to illustrate visible color change. *Rep 2 represents the scatter patterns of microscopic images of colonies presented in this figure. (TIF) [file pone.0105272.s010.tif]

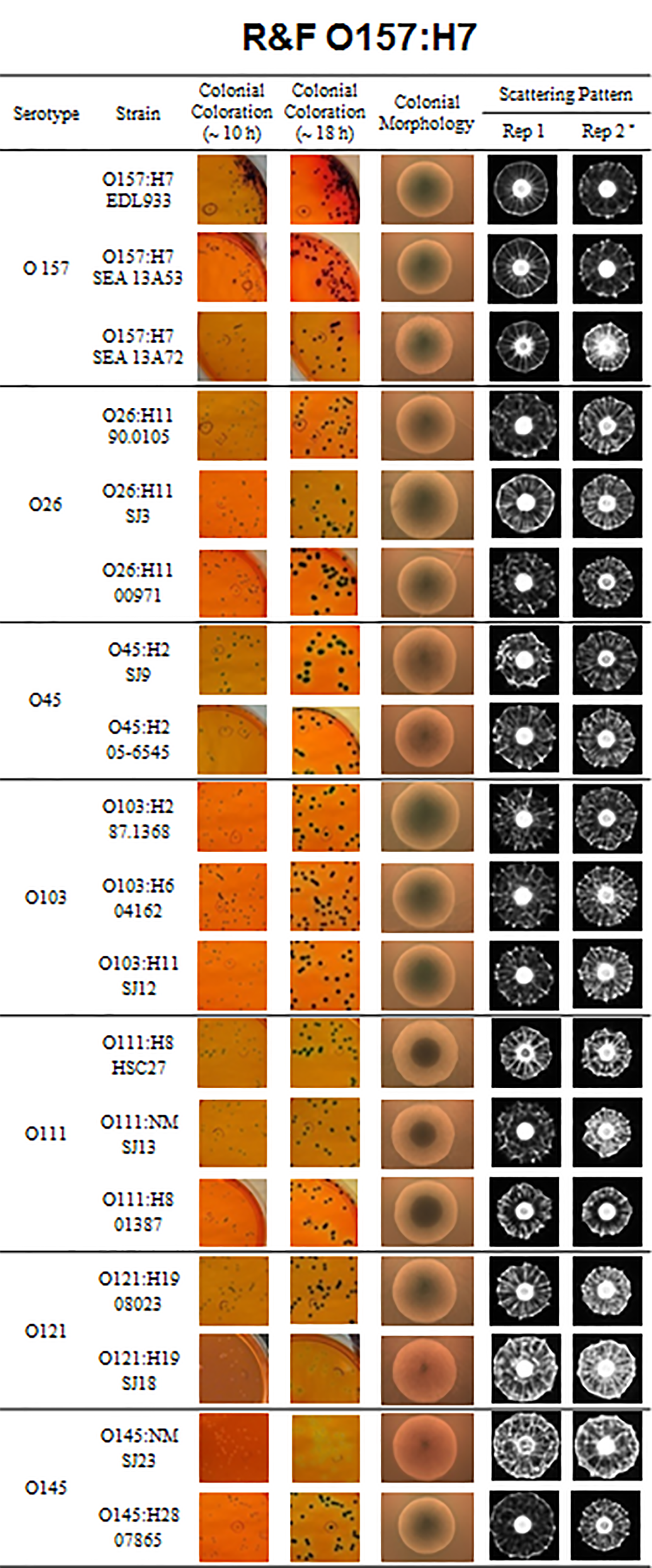

Supplement: Figure S11 — Representative images of colonies on plates, light microscopic images of individual colony and scatter patterns of STEC serovars grown on R&F O157:H7 after about 10 h of incubation at 37°C. Colony images were also captured after 18 h to illustrate visible color change. *Rep 2 represents the scatter patterns of microscopic images of colonies presented in this figure. (TIF) [file pone.0105272.s011.tif]
