# Supplementary material for: Light Scattering Sensor for Direct Identification of Colonies of Escherichia coli Serogroups O26, O45, O103, O111, O121, O145 and O157
Source: PLoS One. 2014 Aug 19;9(8):e105272. doi: 10.1371/journal.pone.0105272 (PMC4138183; doi:10.1371/journal.pone.0105272)

**Table S1.** Oligonucleotide primers used for detection of virulence and O-antigen genes of *E. coli* serovars


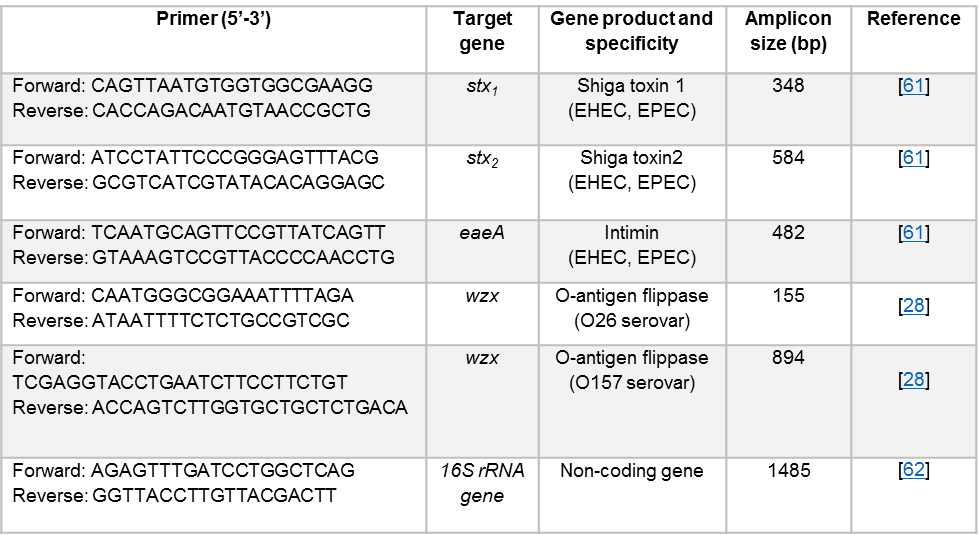

Supplement: Table S1 — Oligonucleotide primers used for detection of virulence and O-antigen genes of E. coli serovars. (DOCX) [file pone.0105272.s012.docx]
